# Attitudes Toward e-Mental Health Services in a Community Sample of Adults: Online Survey

**DOI:** 10.2196/jmir.9109

**Published:** 2018-02-19

**Authors:** Sonja March, Jamin Day, Gabrielle Ritchie, Arlen Rowe, Jeffrey Gough, Tanya Hall, Chin Yan Jackie Yuen, Caroline Leanne Donovan, Michael Ireland

**Affiliations:** ^1^ Institute for Resilient Regions School of Psychology and Counselling University of Southern Queensland Queensland Australia; ^2^ School of Applied Psychology Griffith University Queensland Australia

**Keywords:** eHealth, mHealth, consumer preference, attitude

## Abstract

**Background:**

Despite evidence that e-mental health services are effective, consumer preferences still appear to be in favor of face-to-face services. However, the theory of planned behavior (TPB) suggests that cognitive *intentions* are more proximal to behavior and thus may have a more direct influence on service use. Investigating individual characteristics that influence both preferences and intentions to use e-mental health services is important for better understanding factors that might impede or facilitate the use of these services.

**Objective:**

This study explores predictors of preferences and intentions to access e-mental health services relative to face-to-face services. Five domains were investigated (demographics, technology factors, personality, psychopathology, and beliefs), identified from previous studies and informed by the Internet interventions model. We expected that more participants would report intentions to use e-mental health services relative to reported preferences for this type of support and that these 5 domains would be significantly associated with both intentions and preferences toward online services.

**Methods:**

A mixed sample of 308 community members and university students was recruited through social media and the host institution in Australia. Ages ranged between 17 and 68 years, and 82.5% (254/308) were female. Respondents completed an online survey. Chi-square analysis and *t* tests were used to explore group differences, and logistic regression models were employed to explore factors predicting preferences and intentions.

**Results:**

Most respondents (85.7%, 264/308) preferred face-to-face services over e-mental health services. Relative to preferences, a larger proportion of respondents (39.6%, 122/308) endorsed intentions to use e-mental health services if experiencing mental health difficulties in the future. In terms of the 5 predictor domains, 95% CIs of odds ratios (OR) derived from bootstrapped standard errors suggested that prior experience with online services significantly predicted intentions to use self-help (95% CI 2.08-16.24) and therapist-assisted (95% CI 1.71-11.90) online services in future. Being older predicted increased intentions to use therapist-assisted online services in future (95% CI 1.01-1.06), as did more confidence using computers and the Internet (95% CI 1.06-2.69). Technology confidence was also found to predict greater preference for online services versus face-to-face options (95% CI 1.24-4.82), whereas higher doctor-related locus of control, or LOC (95% CI 0.76-0.95), and extraversion (95% CI 0.88-1.00) were predictive of lower likelihood of preferring online services relative to face-to-face services.

**Conclusions:**

Despite generally low reported preferences toward e-mental health services, intentions to access these services are higher, raising the question of how to best encourage translation of intentions into behavior (ie, actual use of programs). Strategies designed to ease people into new Internet-based mental health programs (to enhance confidence and familiarity) may be important for increasing the likelihood that they will return to such programs later.

## Introduction

### Background

Mental illness presents a significant social and economic burden worldwide, contributing to approximately 13% of the total global burden of disease [1]. Estimated 12-month prevalence rates indicate that as many as one in five adults are likely to currently experience a mental illness, many of whom will not access mental health services or receive treatment [1,2]. Access to mental health care is often limited not only by the well-documented barriers of socioeconomic disadvantage and stigma but also by issues of accessibility, including geographical constraints and cost of services [3].

In an attempt to reduce such barriers to treatment, a number of national and global initiatives have been developed. For instance, the Australian Government has prioritized investment in the development and dissemination of e-mental health services as an alternative for those unable or unwilling to access traditional avenues of support [4]. A number of European nations are also currently working on a joint framework for mental health policy, with a key focus on the implementation of eHealth services in the treatment and prevention of mental illness [5]. A variety of services fall under the term e-mental health, including mental and behavioral health promotion, prevention, treatment and management-oriented interventions that are delivered via the Internet or other electronic technologies, with or without human support [6]. A number of meta-analyses have now shown these services to be comparable to face-to-face options in their effectiveness in treating mental illness [7-9].

Although effective, uptake of e-mental health services remains low [10,11]. For example, in a systematic review of computerized cognitive behavioral therapy (cCBT), Waller and Gilbody [12] reported that only 38% of those recruited into cCBT intervention trials began treatment (median rate), and individuals in cCBT treatments were almost twice as likely to drop out of the intervention as those in active control conditions. Low participation and retention rates have also been observed in other e-mental health service investigations [10]. Furthermore, face-to-face services tend to be viewed more favorably than e-mental health services, with the former rated as more helpful and trustworthy, capable of eliciting better engagement, and viewed more favorably regarding future use [10]. Low preference rates for online services (over face-to-face) are commonly reported, with findings ranging from 1.2% [13] to 29.6% [14] in some studies, whereas face-to-face services tend to have comparably higher rates of preference, ranging from 32.0% [15] to 96.4% [16]. These issues (negative perceptions, poor uptake, and retention rates) are of concern for those investing resources into e-mental health services and highlight the need for a better understanding of the factors that contribute to the use of these services.

The theory of planned behavior (TPB) [17] proposes that behavior can be predicted by intentions, which in turn are partly determined by one’s attitudes toward that behavior. In other words, cognitive intentions theoretically have a more direct influence on behavior than attitudes or preferences, which may be more distal. In a study of young adults, Horgan and Sweeney [14] found that although most participants (79.4%) held a preference for face-to-face support and less than a third (30.8%) had previously used the Internet for mental health information, the majority (68.0%) of the sample indicated they would use the Internet for assistance if required, supporting the notion that preferences and intentions may be related but distinct constructs. Thus, the TPB would argue that greater knowledge of the factors that influence both preferences and intentions toward e-mental health services is important for better understanding the factors that impede or facilitate the use of these services.

The Internet interventions model [18] provides a unifying framework intended to guide the development and improve the understanding of behavior change within online interventions. The model posits that 9 components should be considered for effective development and evaluation of Internet-based treatments, including user characteristics, the environment, support, website characteristics, website use, mechanisms of change, behavior change, symptom improvement, and treatment maintenance. Although the focus of the model is on factors that may influence behavior change and outcomes throughout use of these interventions, it also conceptualizes factors that may contribute to a person’s *use* of Internet interventions; thus, in line with TPB [17], we can infer that these factors may also play a role in shaping intentions and preferences toward e-mental health services. In this study, dual consideration of both the TPB and Internet interventions models provides a framework for identifying and examining potential factors that may influence preferences and intentions toward e-mental health services.

Under *user characteristics*, the Internet interventions model [18] identifies both fixed and modifiable factors that may influence use of, and outcomes from, Internet interventions, including (1) the disease (eg, psychopathology, disease severity, and target problem); (2) demographics (eg, age, gender, and socioeconomic status); (3) traits (eg, personality, temperament, and intelligence); (4) cognitive factors (eg, decision making and developmental stage); (5) beliefs and attitudes (eg, perceived benefits and barriers to treatment); (6) physiological factors (eg, motor functioning); and (7) skills (computer abilities and mindedness). However, the role of such individual characteristics in influencing prospective attitudes toward the use of e-mental health services has not yet been thoroughly tested.

There is little empirical evidence regarding characteristics that influence individuals’ views toward e-mental health services, and the research that does exist is inconsistent [14,19,20-23]. For example, Klein and Cook [20] found that less than 25% of respondents reported a preference for e-mental health services (with or without professional support). These “e-preferers” were more likely to report intentions to use these services in the future and had lower scores on the personality traits of extraversion, agreeableness, emotional stability, and openness to experience. Conversely, Tsan and Day [23] found no relationship between emotional stability (neuroticism) and attitudes toward online help-seeking behavior. Mixed findings are also apparent regarding the influence of attitudes toward technology, prior use of mental health services, and demographic variables (eg, age, gender, education, relationship status, country of birth, and location of residence) on attitudes toward e-mental health services [20,24-26].

We note that some of the inconsistencies may reflect differences across studies in how e-mental health services are defined. For example, there are distinct differences between online services that involve therapist assistance compared with self-help programs without therapist support [10,27], although the former can vary substantially in terms of the amount and type of contact provided. Thus, further investigation into preferences and intentions toward different e-mental health services is warranted. Obtaining a better understanding of individual characteristics that contribute toward these cognitive factors can provide insight useful for triaging or screening patients within routine care settings. Furthermore, such research may assist program developers to create more targeted strategies or tailoring of programs to improve uptake of these services.

### Aims of This Study

This study aimed to explore differences in individual characteristics across both intentions to access and preferences for e-mental health services relative to face-to-face services. For this study, we define e-mental health services as computer-based interventions where the primary content delivery mechanism is through the technology platform (ie, online treatment programs), with or without additional therapist feedback or support. Thus, although important, we exclude telehealth-type services where technology is used to facilitate traditional face-to-face counseling approaches over distance (eg, online counseling using videoconferencing software).

On the basis of the research to date, we expected that participants would prefer face-to-face services over e-mental health services, although we anticipated a higher number of participants to report an intention to use e-mental health services in the future. Considering recent findings that tailored support is strongly preferred over generic programs [19], we also expected that more participants would indicate a greater likelihood of using therapist-assisted e-mental health services in future compared with self-help options.

We also aimed to investigate individual characteristics that might predict attitudes toward accessing e-mental health support (ie, preferences and intentions). We identified 5 general factors that broadly fit within the Internet interventions framework [18] and that have been explored previously with mixed or unclear findings [20,25,26,28,29]. These included the following: (1) *demographics* (ie, age, geographical location, and gender); (2) technology factors including *skills and use* (ie, confidence using computers and the Internet generally, and previous use of e-mental health services); (3) *personality traits* (ie, level of extroversion, neuroticism, or conscientiousness); (4) the *disease* (ie, levels of psychopathology); and (5) *beliefs* regarding influences on mental health outcomes (ie, locus of control, LOC). We expected each of these domains to have a unique influence on both preferences and intentions to use e-mental health services, but given there are mixed findings within the literature, we had no clear a priori hypotheses regarding the direction of effects. In this way, this study was exploratory in nature.

## Methods

### Participants and Procedure

Participants included 308 community members and university students aged between 17 and 68 years (mean 34.26; standard deviation, SD 11.23), who were mostly female (82.5%, 254/308). About half (52.3%, 161/308) lived in regional or remote areas, with the remainder (47.4%, 146/308) residing in major cities (not specified *, n*=1). Most participants reported experiencing either at least one current (51.3%, 158/308) or previous (85.7%, 264/308) mental health concern. These percentages appeared to be above the national average based on previous Australian mental health surveys [30,31], although we did not differentiate between clinical and nonclinical levels of difficulty.

Ethical clearance was obtained from the host institution’s Human Research Ethics Committee (HREC number: H13REA216) before data collection. Recruitment of a community sample from the Australian general public was conducted through promotion on social media and advertisements at the institution’s website. Interested participants were given a Web link for additional information about the study. Participants were required to provide consent via this link and were subsequently directed to the anonymous online battery of questionnaires. As an incentive for participation, community participants were offered the opportunity to enter a draw to win one of three AUD $50 gift vouchers, by submitting their email address separately. Alternatively, undergraduate psychology students enrolled at the host institution were given the option of receiving course credit for an approved undergraduate unit or entering the voucher draw.

### Measures

#### Demographics

Participants were asked to provide their age, gender, and postcode. Individual postcodes were recoded into the Australian Standard Geographical Classification Remoteness Area index categories (RA1, major city; RA2, inner regional; RA3, outer regional; RA4, remote; RA5, very remote) [32], which were then categorized into major city (RA1) or outside major City (RA2-5) for analyses.

#### Technology Factors

##### Confidence With Technology

A single-item measure was used to determine participants’ level of confidence in using computers and the Internet in general on a 5-point scale (1= *very confident* to 5= *really not confident*).

##### Previous Online Help-Seeking Behaviors

A dichotomous (either *yes* or *no*) response item for previous e-mental health service use was created to identify whether or not participants had ever sought help via a therapist-assisted or self-help Internet-based treatment program.

#### Individual Characteristics

##### Locus of Control

The 18-item Multidimensional Health Locus of Control-Form C (MHLC-C) scale [33] was used to assess the degree to which participants attributed their mental health to themselves or to external forces using a 6-point scale (1= *strongly disagree* to 6= *strongly agree)*. The MHLC-C comprises 4 LOC subscales: (1) internal (6 items, eg *, If my condition worsens, it is my own behavior which determines how soon I will feel better again*); (2) external chance (6 items, eg, *As to my condition, what will be will be*); (3) external doctor (3 items, eg, *If I see my doctor regularly, I am less likely to have problems with my condition*); and (4) external others (3 items, eg, *Other people play a big role in whether my condition improves, stays the same, or gets worse*). Previous studies have reported satisfactory reliability for the MHLC-C, with Cronbach alphas in the range of .70-.87 for all subscales [33]. This study observed internal consistencies of alpha=.70 (internal), alpha=.84 (chance), alpha=.68 (doctor), and alpha=.49 (others). As the external (others) subscale had poor reliability in our sample, it was excluded from further analysis.

##### Psychopathology

The 21-item Depression Anxiety Stress Scale [34,35] was used to measure participants’ mental health state over the previous week. The measure comprises three 7-item subscales measuring depression, anxiety, and stress symptoms on a 4-point scale (0= *did not apply to me at all* to 3= *applied to me very much or most of the time*). Previous literature has established adequate internal consistency ranging from .82 to .93 in nonclinical samples [35]. In our study, internal consistency was good (depression: alpha=.92; anxiety: alpha=.85; stress: alpha=.86).

##### Personality

Three subscales from the NEO Five Factor Personality Inventory (NEO-FFI) were used to measure participants’ level of extroversion, neuroticism, and conscientiousness [36]. Together, these subscales comprised 36 items of the full 60-item questionnaire, with 12 items measuring each trait. Responses were recorded on a 5-point scale (1= *strongly disagree or is definitely false* to 5= *strongly agree or is definitely true*). Total subscale scores were calculated by adding items together (with reverse scoring where required), such that higher scores indicate a stronger presence of that trait. Previous research supports the external validity of the NEO-FFI as a measure of adult personality and demonstrates satisfactory internal consistencies for each scale [36,37]. This study demonstrated adequate reliability with Cronbach alphas of .82 (extraversion), .88 (neuroticism), and .88 (conscientiousness).

#### Outcome Variables

##### Service Preference

A single item asked participants to indicate overall, which type of mental health service they would prefer to use if they experienced mental health difficulties in the future: (1) traditional face-to-face mental health assistance (defined as face-to-face therapy with a general practitioner, psychologist, psychiatrist, or counselor); (2) Internet-based mental health assistance with therapist support (eg, support via email, instant messaging, or using video-conferencing); or (3) Internet-based mental health assistance without therapist support. Service preference was converted into a dichotomous variable by combining responses 2 (therapist-supported) and 3 (self-help) into a general Internet-based mental health support category.

##### Intention to Use e-Mental Health Services

Participants were asked about their intended help-seeking behavior should they experience a mental health difficulty in the future. Intention to use a therapist-assisted Internet-based treatment program or a self-help Internet-based treatment program without therapist assistance was measured using an author-developed 5-point scale (1= *extremely likely* to 5= *extremely unlikely*). Responses were later dichotomized (as *yes* or *no*) for analyses; ratings of 1 (*extremely likely*) or 2 (*somewhat likely*) were categorized as “yes,” whereas all other responses (*neither likely or unlikely, somewhat unlikely,* or *extremely unlikely*) were categorized as “no”.

### Statistical Analysis

Analyses were conducted using IBM SPSS Statistics version 22 and R version 3.4.0 [38]. Preliminary exploration of relationships in the data was conducted using zero-order correlations (Spearman rank) for all dependent and independent variables. Descriptive statistics for proportions of participants endorsing preferences and intentions for e-mental health services were explored and compared across demographic characteristics (age, gender, and rural/regional status) using *t* and chi-square tests.

Predictors of preferences and intentions toward online services were examined through logistic regression (LR) analyses. Given the exploratory nature of the study and the large number of predictors included, our 5 predictor groupings (demographics, technology factors, LOC, psychopathology, and personality factors) were first tested individually through a series of LR models for each of the 3 dependent variables (service preference, intentions to use therapist-assisted e-mental health services, and intentions to use self-help e-mental health services). Significant demographic variables from the first LR model were carried through into each subsequent model as covariates to examine the influence of psychological and technological factors after removing variation attributable to demographic differences.

We then examined a final model for each dependent variable, incorporating all significant individual predictors from prior LR groupings (based on evaluation of 95% CI) to evaluate their relative contribution in the context of other predictive domains. Variance inflation factors were reviewed for all individual and combined models to check for multicollinearity, with no issues identified. Minimum sample size recommendations generally suggest at least 10-20 events per predictor for LRs [39,40], with events defined as the proportion of cases in the least frequent of the two outcome categories. As some dichotomous response categories were endorsed by relatively few respondents, to account for potential problems arising from too many predictors in the final combined models (eg, biased estimates), we used nonparametric bootstrapping techniques to estimate bias-corrected confidence intervals (CIs), in line with suggestions by Vittinghoff and McCulloch [40]. Parameter estimates and bias-corrected CIs were obtained for these models using 10,000 resamples of the data.

For all final combined models, analyses were run both with and without the bootstrapping method to check if there were any substantive differences in terms of which predictor variables were significant or nonsignificant. No differences were evident, and thus the results from bootstrapping analyses are presented.

## Results

### Preliminary Analyses

The most common mental health concerns reported by participants were stress (40.9%, 126/308), anxiety (28.9%, 89/308), and depression (22.7%, 70/308). Most participants reported being either confident (28.9%, 89/308) or very confident (65.9%, 203/308) using computers and the Internet. There were no demographic differences between those who were confident/very confident and those who were not. Only a small proportion of participants had previously sought help through e-mental health services (6.8%, 21/308).

Spearman rank correlations between all variables are shown in Table 1. Among the 3 dependent variables, service preference had a low correlation with intention to use both self-help and therapist-assisted online services (both *r*_s_<.26), whereas both intention ratings were moderately correlated with each other (*r*_s_=.52). All zero-order correlations of independent variables with intentions and preference were low (*r*_s_<.30), although some were significant.

Regarding demographic variables, participant age was associated with the most other variables, suggesting that younger participants were more likely to report less desirable characteristics (eg, psychopathology: *r*_s_=−.12 to −.21; neuroticism: *r*_s_=−.17, and chance LOC: *r*_s_=−.26), but were also more confident with computers and the Internet (*r*_s_=−.23). Older participants tended to score higher on conscientiousness (*r*_s_=.16) and internal LOC (*r*_s_=.17).

### Proportion of Respondents Preferring and Intending to Use e-Mental Health Services

Consistent with our hypotheses, the majority of respondents (85.7%, 264/308) indicated a preference for traditional face-to-face support. A total of 10.7% (33/308) respondents preferred therapist-supported online interventions and only 3.6% (11/308) preferred self-directed online support. As outlined above, the latter two categories were combined into a single e-mental health preference category (14.3%, 44/308). Using this dichotomized variable, there were no significant differences in age, *t*_306_=0.58, *P*=.57, or gender, χ^2^_1_=0.1, *P*=.76, between those who preferred face-to-face and those who preferred Internet-based services. Service preference was significantly different across geographic locations (metropolitan vs. nonmetropolitan), χ^2^_1_=5.3, *P*=.02, with a greater proportion of metropolitan participants (19.2%, 28/146) compared with nonmetropolitan participants (9.9%, 16/161) preferring Internet-based services.

With regard to intentions, 25.0% (77/308) of participants indicated they would be “likely” or “very likely” to use self-help e-mental health services in the future if experiencing mental health difficulties. There were no differences in age, *t*_306_=1.31, *P*=.19; gender, χ^2^_1_=−0.8, *P*=.39; or location, χ^2^_1_=0.2, *P*=.67, between participants who reported intentions to use these services and those who did not. A larger proportion of participants reported intentions to use therapist-assisted e-mental health services in the future (33.8%, 104/308). These respondents were slightly older (mean 36.64, SD 10.77) than those who did not intend to use these services (mean 33.04, SD 11.28), *t*_306_=−2.69, *P=*.007. There were no significant differences in gender, χ^2^_1_=0.2, *P*=.70, or location, χ^2^_1_=0.1, *P*=.73. Single-group chi-square analysis indicated that the proportion of respondents responding they would use therapist-assisted services was significantly greater than the proportion likely to use self-help services, χ^2^_1_=12.6, *P*<.001. There was some overlap between these two categories, with 39.6% (122/308) endorsing future use of self-help and/or therapist-assisted services if needed.

Of the 308 survey respondents, 96.4% (297/308) indicated they would be "likely" or "very likely" to use either e-mental health or face-to-face services if experiencing mental health problems in future. Examining these responses, the proportion stating they would be likely to *use* online services (self-help and/or therapist-assisted; 39.6%, 122/308) was significantly greater than the relative proportion reporting a *preference* for online services (14.3%, 44/308), χ^2^_1_=177.5, *P*<.001. Follow-up cross-tabulation of these two response variables showed that for those preferring traditional face-to-face support approaches, more than a third (34.1%, 90/264) still indicated they would be likely to use online services in future. For those that preferred online services, most (76%, 32/44) indicated they would also be likely to use them.

**Table 1:** Spearman rank correlations for variables used in logistic regression analyses. SH: self-help online (intention to use). TH: therapist-supported online (intention to use). DASS-21: Depression Anxiety Stress Scale-21.

Predictors	1	2	3	4	5	6	7	8	9	10	11	12	13	14	15	16	17
1. Service preference	1																
2. SH	.26^a^	1															
3. TH	.20^a^	.52^a^	1														
4. Gender	.02	.05	.02	1													
5. Age	.05	.09	.16^a^	−.02	1												
6. Location	−.13^b^	.02	.02	.13^b^	.12^b^	1											
7. Online confidence	.11	.11	.10	−.01	−.23^a^	−.12^b^	1										
8. Prior use	.15^a^	.20^a^	.22^a^	−.05	.01	−.05	.01	1									
9. LOC (I)^c^	−.06	−.06	−.02	−.09	.17^a^	−.08	−.04	−.04	1								
10. LOC (C)^d^	.06	.04	−.08	−.08	−.26^a^	−.07	−.07	.08	−.10	1							
11. LOC (D)^i^	−.21^a^	−.11	−.01	−05	−.02	.08	.03	−.01	.06	−.06	1						
12. DASS-21 depression	.15^a^	.03	−.08	−.14^b^	−.15^a^	−.11	−.07	.11^b^	.12^b^	.31^a^	−.06	1					
13. DASS-21 anxiety	−.03	.00	−.07	−.04	−.21^a^	−.01	−.11	.09	.04	.30^a^	.03	.64^a^	1				
14. DASS-21 stress	.08	.05	−.05	.06	−.12^b^	−.05	−.05	.14^b^	.06	.24^a^	−.09	.70^a^	.67^a^	1			
15. Neuroticism	.15^a^	.03	−.10	.03	−.17^a^	−.04	−.10	.09	.04	.27^a^	−.03	.71^a^	.61^a^	.66^a^	1		
16. Extraversion	−.18^a^	−.01	.01	.06	−.01	.07	.14^b^	−.08	−.12^b^	−.21^a^	.13^b^	−.44^a^	−.28^a^	−.31^a^	−.42^a^	1	
17. Conscientiousness	−.10	.08	.04	.12^b^	.16^a^	−.01	.05	−.07	−.06	−.16^a^	.05	−.36^a^	−.17^a^	−.09	−.32^a^	.34^a^	1

^a^Significant at the *P*<.01 level.

^b^Significant at the *P*<.05 level.

^c^LOC (I): locus of control—internal.

^d^LOC (C): locus of control—chance.

^e^LOC (D): locus of control—doctor.

### Predictors of Service Preferences

We first report results of the initial LR analyses for the 5 variable groupings (see Table 2), followed by the combined model where significant predictors were retained (see Table 3).

For demographic variables, the overall model was nonsignificant, χ^2^_3_=6.6, *P*=.09, Cox and Snell *R*^2^ (*R*^2^_CS_)=.02, Nagelkerke *R*^2^ (*R*^2^_N_)=.04. However, location (ie, major city vs outside major city) was a significant individual predictor of service preference, *P*=.02. This discrepancy may be due to a masking effect from the inclusion of multiple nonsignificant predictors in the model. As earlier chi-square comparison of service preference across location was also significant, we decided to retain location as a covariate within subsequent models to control for potential confounding effects when exploring other individual factors.

For technology factors, the model was significant, χ^2^_3_=14.0, *P*=.003, *R*^2^_CS_=.05, *R*^2^_N_=.08. Controlling for location, previous use of online mental health services significantly predicted service preference (*P*=.02), with the likelihood of preferring e-mental health services estimated as three times greater for those who had used these services in the past. Online confidence was nonsignificant based on *P* value, but was significant based on its CIs, odds ratio (OR) 1.85 (95% CI 1.01-3.89), *P*=.07. We note here that by default, R computes *P* values using a Wald test, whereas 95% CIs are computed using the likelihood ratio test. At large sample sizes, these tests are asymptotically equivalent; however, the likelihood ratio test is generally considered to perform better in smaller samples as the Wald test becomes too conservative [39,41]. As such, we retained both technology predictors for further analysis in the combined model.

Three LOC subscales (*doctor*, *chance*, and *internal*) were entered together into a third LR model along with the location covariate. The overall model was significant, χ^2^_4_=18.8, *P*=.001, *R*^2^_CS_=.06, *R*^2^_N_=.11. Of the 3 LOC subscales, doctor LOC was a significant predictor of service preference (*P*=.001), indicating that for every one-unit increase in the doctor-related external LOC (ie, potential for change is attributed to the influence of doctors), participants were 16% less likely to prefer Internet-based mental health services. Chance and internal LOC subscales were not significant independent predictors.

**Table 2:** Logistic regression analyses for preference for online services over face-to-face, intentions to use therapist-assisted e-mental health services, and intentions to use self-help e-mental health services.

Predictors		Preference (online vs face-to-face)			Intention: online therapist-assisted services^a^			Intention: online self-help services			
		*B*^b^	SE	OR^c^ (95% CI)	*B*	SE	OR (95% CI)	*B*		SE	OR (95% CI)
**Demographic variables**^d^ **(n=307)**											
	Gender: female	0.31	0.45	1.36 (0.59-3.56)	0.18	0.33	1.19 (0.63-2.32)	0.34		0.37	1.4 (0.7-3.03)
	Age^e^	0.14	0.15	1.01 (0.99-1.04)	0.03^f^	0.01	1.03 (1.01-1.05)	0.02		0.01	1.02 (0.99-1.04)
	Location: major city^a^	0.84^g^	0.35	2.31 (1.19-4.63)	0.01	0.25	1.01 (0.62-1.65)	−0.04		0.27	0.96 (0.57-1.64)
**Technology variables (n=308)**											
	Online confidence	0.61	0.34	1.85 (1.01-3.89)	0.51^g^	0.22	1.67 (1.1-2.64)	0.46		0.24	1.59 (1.01-2.65)
	Previous use: yes	1.16^g^	0.51	3.2 (1.13-8.42)	1.73^e^	0.51	5.67 (2.2-16.51)	1.50^f^		0.47	4.5 (1.82-11.54)
**Locus of control**^h^ **(n=301)**											
	Internal	−0.04	0.04	0.97 (0.9-1.04)	−0.02	0.03	0.98 (0.93-1.03)	−0.02		0.03	0.98 (0.93-1.03)
	Chance	0.03	0.03	1.03 (0.97-1.09)	−0.02	0.02	0.98 (0.93-1.02)	0.01		0.02	1.01 (0.96-1.06)
	Doctor	−0.17^f^	0.05	0.84 (0.76-0.93)	0.00	0.04	1 (0.93-1.08)	−0.08		0.04	0.93 (0.85-1)
**Psychopathology**^h^ **(n=308)**											
	Depression	0.14^g^	0.06	1.15 (1.03-1.3)	−0.03	0.05	0.97 (0.88-1.06)	−0.04		0.05	0.96 (0.88-1.06)
	Anxiety	−0.14^g^	0.07	0.87 (0.75-0.99)	−0.01	0.05	0.99 (0.89-1.09)	0.03		0.05	1.03 (0.93-1.15)
	Stress	0.03	0.06	1.03 (0.91-1.15)	0.01	0.05	1.01 (0.92-1.1)	0.03		0.05	1.03 (0.93-1.13)
**Personality**^h^ **(n=308)**											
	Extraversion	−0.05^g^	0.03	0.95 (0.9-1)	−0.01	0.02	0.99 (0.95-1.03)	0.00		0.02	1 (0.96-1.04)
	Neuroticism	0.02	0.02	1.02 (0.98-1.07)	−0.02	0.02	0.98 (0.95-1.01)	0.02		0.02	1.02 (0.98-1.05)
	Conscientiousness	−0.02	0.02	0.98 (0.94-1.03)	0.00	0.02	1 (0.97-1.04)	0.03		0.02	1.03 (0.99-1.07)

^a^Retained as covariate in all subsequent *preference* models.

^b^B: coefficient.

^c^OR: odds ratio.

^d^Overall model fit not significant at the *P*<.05 level for dependent variables *preference*, *therapist-assisted intentions,* and *self-help intentions*.

^e^Retained as covariate in all subsequent *therapist-assisted intentions* models.

^f^Significant at the *P*<.01 level.

^g^Significant at the *P*<.05 level.

^h^Overall model fit not significant at the *P*<.05 level for dependent variables *therapist-assisted intentions* and *self-help intentions*.

For psychopathology, the overall model was significant, χ^2^_4_=13.9, *P*=.008, *R*^2^_CS_=.04, *R*^2^_N_=.08. Both depression (*P*=.02) and anxiety (*P*=.04) were significant individual predictors of service preference. For every one-unit increase in depression scores, participants were 15% more likely to prefer Internet-based mental health services; conversely, for every one-unit increase in anxiety scores, participants were 13% less likely to report a preference for Internet-based services. Stress was not significant in this model.

The overall model for personality factors including neuroticism, extraversion, and conscientiousness from the NEO-FFI was significant, χ^2^_4_=17.6, *P*=.002, *R*^2^_CS_=.06, *R*^2^_N_=.10. Of the three personality factors, extraversion was the only significant predictor of service preference (*P*=.04); for every one-unit increase in extraversion, there was a 5% lower likelihood of preferring Internet-based mental health services.

#### Combined Model

As outlined above, the following variables were entered together into a single LR to examine their combined and relative contribution toward service preference: location, online confidence, previous use of online services, external LOC (doctor), depression, anxiety, and extraversion. The model fit was significant, χ^2^_7_=38.9, *P*<.001, *R*^2^_CS_=.12, *R*^2^_N_=.22. Bootstrapped coefficients and bias-corrected CIs are shown in Table 3.

**Table 3:** Logistic regression analyses for combined variables predicting service preference, intention to use therapist-assisted e-mental health services, and intention to use self-help e-mental health services.

Predictor		*B* (coefficient)	Standard error	Odds ratio (95% CI)^a^
**Service preference (n=300)**				
	Location: major city	0.48	0.39	1.62 (0.76-3.41)
	Online confidence	0.96^b^	0.37	2.62 (1.24-4.82)
	Previous use: yes	0.97	0.88	2.63 (0.60-9.23)
	LOC doctor^c^	−0.17^b^	0.88	0.84 (0.76-0.95)
	Depression	0.12	0.07	1.12 (0.98-1.28)
	Anxiety	−0.15	0.09	0.86 (0.74-1.04)
	Extraversion	−0.07^b^	0.03	0.93 (0.88-1.00)
**Therapist-assisted online services (n=308)**				
	Age	0.04^b^	0.01	1.04 (1.01-1.06)
	Previous use: yes	1.83^b^	0.77	6.22 (2.08-16.24)
	Online confidence	0.54^b^	0.24	1.71 (1.06-2.69)
**Self-help online services (n=308)**				
	Previous use: yes	1.54^b^	0.50	4.65 (1.71-11.90)
	Online confidence	0.50	0.26	1.64 (0.97-2.68)

^a^Nonparametric bootstrapping was used to compute coefficients and bias-corrected CIs.

^b^Significant coefficient (*B*) based on 95% CI for exp(*B*) (ie, odds ratio).

^c^LOC: locus of control.

Significant predictors in the full model were external LOC (doctor), extraversion, and confidence with computers and the Internet. As participants scored higher on extraversion or LOC was more strongly oriented toward their doctor, they became significantly less likely to endorse a preference for online services. Conversely, preference for online services became significantly more likely as online confidence increased.

### Predictors of Intention to Access Therapist-Assisted e-Mental Health Services

As with preferences, the overall LR model for demographic variables predicting intentions to use therapist-assisted e-mental health services in future was nonsignificant, χ^2^_3_=7.6, *P*=.056, *R*^2^_CS_=.02, *R*^2^_N_=.03. However, given that participant age was a significant individual predictor (*P*=.007), and earlier *t* tests showed significant age differences between those that did and did not intend to use therapist-assisted services, we retained it as a covariate for subsequent models.

Models involving LOC (χ^2^_4_=6.7, *P*=.16, *R*^2^_CS_=.02, *R*^2^_N_=.03), psychopathology (χ^2^_4_=8.6, *P*=.07, *R*^2^_CS_=.03, *R*^2^_N_=.04), and personality (χ^2^_4_=8.9, *P*=.06, *R*^2^_CS_=.03, *R*^2^_N_=.04) were not significant. However, the model for technology factors, with age included as covariate, was significant, χ^2^_3_=26.8, *P*<.001, *R*^2^_CS_=.08, *R*^2^_N_=.12. Online confidence (*P*=.02) and prior use (*P*<.001) were both significant individual predictors, indicating a greater likelihood of intending to use therapist-assisted e-mental health services as these increased.

#### Combined Model

Bootstrapped estimates were computed for the model containing age, online confidence, and prior use. Results are shown in Table 3. Age remained a significant predictor of intentions, as did prior use of online services and online confidence. Participants were over six times more likely to report they intended to use therapist-assisted online mental health services in future if they had used similar services previously, and 71% more likely to report intention to use these services for each unit increase in confidence.

### Predictors of Intention to Access Self-Help e-Mental Health Services

For the prediction of intentions to use self-help online interventions, the LR models containing demographics (χ^2^_3_=2.7, *P*=.44, *R*^2^_CS_=.01, *R*^2^_N_=.01), LOC (χ^2^_3_=4.6, *P*=.21, *R*^2^_CS_=.02, *R*^2^_N_=.02), psychopathology (χ^2^_3_=1.1, *P*=.79, *R*^2^_CS_<.01, *R*^2^_N_=.01), and personality (χ^2^_3_=3.3, *P*=.35, *R*^2^_CS_=.01, *R*^2^_N_=.02) were not significant. Parameter estimates are shown in Table 2. There were no significant individual predictors from each of these models at the *P*<.05 level.

For technology factors, the overall model was significant, χ^2^_2_=14.7, *P*=.001, *R*^2^_CS_=.05, *R*^2^_N_=.07. Previous use of online mental health services was a significant predictor (*P*=.001), indicating that the likelihood of reporting an intention to use self-help e-mental health services in future was more than four times higher for those with prior experience using similar online services. Confidence with computers and the Internet was not significant based on estimated *P* value; however, CIs indicated that retaining these factors in the combined model could be valuable, OR 1.59 (95% CI 1.01-2.65), *P*=.06.

#### Combined Model

A model containing previous online service use and technology confidence was estimated using bootstrapping, with estimates shown in Table 3. CIs for online confidence no longer indicated significance, whereas previous use of online services remained a significant predictor.

## Discussion

### Principal Findings

This study examined attitudes towards e-mental health services in a community and student sample of adults, with a focus on both individual preferences and intentions regarding the use of these services in future. Most participants in our sample endorsed a preference for face-to-face treatment over Internet-based options with or without support, with similar proportions as identified in prior research [14]. One-quarter (self-help) to one-third (therapist-assisted) of the overall sample indicated they would use online services in future if experiencing mental health difficulties, and this rate was stable (35.3%) when examining only participants who reported a preference for face-to-face services, which represented the majority of our sample.

Overall, our findings supported our first set of hypotheses, specifically, that preferences for e-mental health services appear to be somewhat distinct from participants’ views regarding the likelihood that they would use these services, with individuals reporting greater intentions to use them than what is reflected in their reported preferences. These findings are also largely in line with results from recent studies with adults and students regarding attitudes toward e-mental health services, where findings indicate that intention to use online services ranges between 22% and 70.8% [14,19,20,42]. The highest rates in previous studies were with student populations or where the reason for help-seeking was a diagnosed mental health condition. In community samples, and where the reason for help-seeking was less severe difficulties (eg, mental health concerns), rates more closely reflect those reported in our study.

We further examined predictors of preferences and intentions toward online services across a broad range of domains, drawing on constructs from the Internet interventions model [18], including demographic, personality, psychopathology, attributional, and technological factors. We found only partial support for these individual characteristics as predictors of preference and intentions toward the prospective use of online treatment services. Living outside of a major city was associated with a lower preference for online services, as was less confidence with computers and the Internet, and not having used online services in the past. The fact that those facing more geographical restrictions were also more likely to prefer face-to-face services is noteworthy, as advances in e-mental health services are intended to improve access to services for those with geographical restrictions [43]. There is some evidence from previous literature that those in geographically remote areas tend to also have less access to technology, and thus may have lower levels of computer confidence [44-46]. This is reflected in our combined preferences model, where online confidence remained significant but location and previous use did not. This likely indicates some shared variance between the 3 variables in predicting preferences, with confidence having the stronger effect. Thus, increasing the availability of e-mental health services alone may not actually circumvent barriers associated with regional access, especially if those living in these areas lack the skills or confidence to try them.

Other variables associated with the likelihood of preferring face-to-face services were doctor-related LOC, anxiety, depression, and extraversion, with only doctor LOC and extraversion significant in the combined model, predicting a lower likelihood of preferring e-mental health services. Doctor LOC reflects a tendency to attribute health outcomes externally to the influence of health professionals. Findings are mixed in the broader literature regarding the impact of external versus internal LOC on treatment preferences, although those with a more external LOC may do better with interventions that provide greater structure or contact [47]. If e-mental health services are viewed as more unstructured than face-to-face therapy, this could be a deterrent for some people who prefer more directive approaches. We found, however, that after controlling for other factors such as technology confidence, doctor LOC predicted support preferences only, with no bearing on reported intentions to use services. These findings are encouraging, as they suggest that despite some stable individual characteristics being associated with a person’s support preferences, changeable factors such as increasing access, familiarity, and providing education around e-mental health programs are likely to have a greater impact on intentions and subsequent use of online services, even when these options are less preferred.

When examining psychopathology, we found no relation to intentions to use e-mental health services. In the individual models for psychopathology, there was some evidence that individuals with higher levels of depression were more likely to prefer online supports, whereas those with higher levels of anxiety were more likely to prefer face-to-face options, although these were no longer significant in the combined model when controlling for other factors. Further research within a clinical sample may be warranted to see whether these findings are generalizable, an issue we discuss further below.

Age, prior use, and online confidence were significantly associated with intention to use online therapist-assisted services in future if experiencing mental health difficulties. It has been found that younger people are less likely to seek help for mental health problems in general, although they are increasingly turning to self-help Internet-based options [48]. Our findings suggest that younger participants may hold similar negative views regarding therapist-supported online services to those they hold around face-to-face services (eg, concerns about stigma, wanting to rely on self), leading to lower intentions to use therapist-supported online services in future. Self-directed services, however, did not show this effect, and may present a viable target for improving e-mental health service use among younger people.

For intention to use self-help online services, prior use and online confidence were significant individual predictors, although only prior use was significant in the combined model. Conservatively speaking, ORs suggested individuals were at least around twice as likely to endorse the use of online services in future if they have prior experience with similar services. We note there were large CIs for these estimates, likely a result of the small proportion of our sample reporting prior use of e-mental health services (6.8%, 21/308). Nonetheless, the fact that prior use was consistently important throughout our study, and related to both preferences and intentions, suggests it is an important target for intervention strategies moving forward. It may be useful, for example, to target people who have already used e-mental health services (early responders) and who are again seeking help for mental health difficulties, or to provide notifications of program updates or new releases to users who have previously tried an online intervention but dropped out. Additionally, easing people in through low barrier, low-intensity services that are easy to engage with may increase the chances of engagement with more comprehensive offerings in future should the need arise.

### Limitations

Although the Internet interventions model [18] described earlier was used as a broad framework within this study for identifying individual user characteristics that may predict preferences and attitudes toward Internet interventions, it is important to contextualize these findings. First, the Internet interventions model aims to provide a framework for understanding use and outcomes of Internet interventions, in other words, how online treatments lead to change and how their efficacy can be maximized. Respondents in our study did not partake in any online treatments, and thus data are limited to attitudes and intentions only. As exemplified by the TPB framework [17], behaviors (eg, engagement with online treatment) can be viewed as distinct from how someone views online treatment. Although there has been some prior work demonstrating a relationship between individual characteristics and willingness to participate in online treatments [49], whether these same characteristics equally predict later effective engagement is not clear. This same limitation applies to this study. Longitudinal monitoring of how well predictors of attitudes and intentions toward online treatments translate into the subsequent uptake of services appears to be an opportunity for further exploration.

Second, our study was intentionally broad in terms of assessing intentions toward online treatment for general mental health difficulties and was conducted with a community sample where not all participants were experiencing current mental health difficulties or were actively seeking treatment. Individual characteristics proposed within the Internet interventions [18] model—for example, elevated levels of depression or anxiety (ie, the severity of the problem)—may be more predictive of attitudes toward targeted online treatment programs that hold relevance for someone experiencing a specific mental health difficulty. As such, assessing attitudes and intentions toward online treatment within a clinical sample of respondents may yield different findings regarding the influence of psychopathology.

Third, reliance on a forced choice for the preference construct means that we were unable to determine the strength of the preference toward either service type and does not allow us to directly compare preferences to intentions. Future research should examine the strength of preferences and intentions using similar rating scales to enable more direct comparison. Other limitations include the representativeness of the sample, which consisted mostly of females, confident computer users, and people who endorsed a higher than average number of mental health concerns, and so the generalizability of results may be limited. Finally, some elements of the Internet interventions model (eg, cognitive and physiological factors; [18]) were not incorporated in our study, and we were unable to assess external LOC pertaining to others due to poor reliability of the subscale in our sample.

### Conclusions

In summary, we found low rates of preference for online services compared with face-to-face treatments, and this was more evident for those living outside of major cities, where these services are intended to improve reach. Despite low preferences, intentions to access these services remain promising, raising the question of how to best encourage translation of intentions into behavior (ie, actual use of services). The importance of being confident with computers and the Internet suggests that preferences may undergo a natural shift toward e-mental health services over time, as access to technology continues to increase in regional areas and as young people continue to grow up as digital natives. However, encouraging early use of brief online programs may also be an effective strategy that could enhance uptake of future e-mental health programs. Integrating programs into nonthreatening contexts (eg, schools, primary care) may be one way to provide people with a taste of these options and increase the chances they will try them later. Health care professionals may play a role here, through promoting evidence-based online treatment programs, encouraging patients to try online services following discharge from face-to-face care, or providing in-person demonstrations or brief trial-runs of online programs in clinics or waiting rooms, to both normalize and demystify these services. This study adds to the emerging literature on consumer attitudes toward e-mental health services through examining preferences and intentions; however, more directed research around how these translate into use of e-mental health services is needed.

## Abbreviations

cCBT: computerized cognitive behavioral therapy
LOC: locus of control
LR: logistic regression
MHLC-C: multidimensional health locus of control—form C
NEO-FFI: NEO five-factor personality inventory
OR: odds ratio
TPB: theory of planned behavior
